# Protocol for evaluation of the feasibility and preliminary efficacy of a targeted transition readiness workshop intervention for pediatric brain tumor survivors

**DOI:** 10.1186/s40814-023-01437-5

**Published:** 2024-01-19

**Authors:** Marco Bonanno, Leandra Desjardins, Tziona Lugasi, Julie Carrier, Nathalie Labonté, Serge Sultan, Hallie Coltin, Sébastien Perrault, Carole Provost, Caroline Laverdière, Nancy Cloutier, Andrea Saragosti, Émilie Régnier-Trudeau, Benedicte Koukoui

**Affiliations:** 1 Hematology-Oncology Unit, Sainte-Justine University Health Center, Montreal, QC Canada; 2 Sainte-Justine Research Health Center, Montreal, QC Canada; 3 Department of Psychology, Université de Montréal, Montreal, Québec Canada; 4 École Des Petits-ExpCrateurs, Marie-Victorin School Board, Montreal, QC Canada; 5 Department of Pediatrics, Université de Montréal, Montreal, Québec Canada

**Keywords:** Pediatric brain tumor survivors, Implementation tool, Pilot project, Feasibility studies, Transition, Patient-centered intervention, Parent support, Mixed method study

## Abstract

**Background:**

Pediatric brain tumor survivors (PBTS) are at risk of physical, cognitive, and psychosocial challenges related to their diagnosis and treatment. Routine follow-up care as adults is therefore essential to their long-term health and quality of life. In order to successfully navigate to adult healthcare, it is recommended that youth develop transition readiness skills. Existing transition readiness interventions often focus on disease management. However, PBTS are also at risk of social competence and cognitive functioning challenges. In this paper, we describe the protocol of this pilot study and the methodology that will be used for the evaluation of the feasibility, acceptability, and preliminary efficacy testing of the first targeted transition intervention workshops specifically designed to meet the needs of PBTS and their caregivers.

**Methods:**

This study will use a mixed method to evaluate three 1 ½-h workshops targeted for dyads (*N* = 40) of PBTS (14 years or older) and their parents. Dyads will be recruited via a community pediatric cancer organization and the long-term follow-up clinic of a large pediatric hospital. Participants will complete an online survey which includes the Transition Readiness Assessment Questionnaire (TRAQ) before and after the workshops.

Each workshop will cover a specific topic related to PBTS transition readiness: disease management, social competence, and cognitive functioning. Workshops will follow the same structure: topic presentation, discussion by a post-transfer survivor or parent, teaching two strategies, and workshop evaluation. Workshops will be co-led by healthcare specialists and patient partners.

Feasibility and acceptability will be assessed via recruitment, attendance, retention, and Likert scales, and they will be analyzed by describing and comparing rates. Satisfaction will be measured using satisfaction surveys and audio-recorded focus groups. Qualitative data will be described through thematic content analysis. In order to test the preliminary efficacy of this study, we will compare transition readiness skills pre- and post-workshops using paired samples *T* test and ANCOVA to examine the impact of workshop on TRAQ skills.

**Discussion:**

Results of the study will inform refinement and future broader implementation of targeted transition readiness workshops for the specific needs of pediatric brain tumor survivors.

## Introduction

### Background

#### Pediatric Brain Tumor Survivor population (PBTS)

Brain tumors are the second most common form of pediatric cancer, and the majority (75%) will survive their disease [1, 2]. Treatment often involves craniosurgery and intensive therapy such as cranial radiation and chemotherapy. As a result of their tumor and treatment, PBTS are particularly vulnerable to late effects which may include physical, cognitive, and psychosocial difficulties (Perreault, Desjardins, Scheinemann, accepted). Given these risks, it is essential PBTS continue to receive lifelong routine follow-up medical care, beyond the pediatric treatment setting. Unfortunately, most PBTS do not receive the preparatory guidance necessary to support their successful transition from pediatric to adult healthcare [3, 4]. Lack of follow-up care in adult settings can have devastating consequences, putting individuals at risk for impairment, secondary cancers, stroke, and premature death [5].

#### Transition

Transition from pediatric to adult sector is an essential process supporting youth in acquiring self-care, self-advocacy, decision-making skills, and the knowledge necessary to pursue care in adult healthcare settings [6, 7]. Transition is a protracted process which can be distinguished from the discrete point of transfer, when an individual no longer receives care in a pediatric setting and can begin receiving care from adult healthcare services. Successful transition is crucial to lifelong health outcomes for individuals with pediatric-onset chronic conditions such as cancer. Unfortunately, the majority of youth with childhood-onset chronic conditions do not receive the necessary preparation for transition to adult care [8].

#### Transition readiness

Guidelines for supporting transition in clinical practice indicate that core elements of transition care involve transition tracking and monitoring, assessment of transition readiness, and transition planning [9]. Despite the availability of transition measures, only 8% of transition care providers use transition assessments in their practice [10]. A well-validated, disease-neutral, and multi-informant measure that has received the strongest empirical support in the assessment of transition readiness skills is the Transition Readiness Assessment Questionnaire (TRAQ) [11–13]. Examples of transition readiness skills assessed by the TRAQ include “Do you make a list of questions before the doctor’s visit?” and “Do you fill a prescription if you need to?” [12]. In addition to assessing transition skills assessments, key transition planning and preparation skills involve disease education and transition skill building (e.g., planning own medical appointments) [14]. Current recommendations are to begin transition skill building in early adolescence, which is a pivotal time in broader youth development. In addition to preparing to transition from pediatric to adult care, young people are also transitioning from adolescence to adulthood [15, 16], which involves the development of autonomy and personal identity as it relates to choices in employment and social integration in general [17, 18].

#### PBTS and transition

Among pediatric cancer survivors, PBTS are often noted to have greater deficits in three specific areas impacting their transition readiness: disease self-management, social competence, and cognitive functioning [17–19]. These areas have been identified as significant barriers to a successful transfer [10, 20]. PBTS are often unprepared for the abrupt shift to adult care where they are required to be solely responsible for their medical care, including planning and attending medical appointments alone. Studies show that PBTS have more difficulties compared to other pediatric populations in independently managing symptoms from their illness, which is essential for longer-term autonomous participation in adult healthcare [21, 22]. Moreover, parental overprotection has been a frequently noted barrier to transition that can prevent or slows down disease management in the PBTS population [23–25]. PBTS also experience greater challenges in social competence, which may impede PBTS from attending and fully participating in adult care medical appointments (e.g., asking questions, participating independently) [26]. Finally, PBTS are at risk of neurocognitive sequelae, impacting their academic achievement and long-term employment, and often lack the knowledge necessary to actively address these challenges [22, 27]. Cognitive challenges may impact transition readiness skills, such as the ability to keep track of appointments, take medication, and remember information provided during appointments. Overall, there is a pressing need for multifaceted transition interventions to support the transition readiness of PBTS.

Existing limited applications of transition care interventions have often focused on disease self-management skills, with the same intervention often applied across various pediatric populations [14, 28]. Unfortunately, this uniform approach erodes the importance of addressing the specific transition needs of PBTS and other pediatric chronic and degenerative disease populations as well, who can present major social competences difficulties and important neurocognitive sequelae. Failing to address these specific challenges when preparing PBTS for the transition to adult sector can significantly decrease their readiness and undermine their participation in health follow-up services offered in adult settings [6, 29].

There have been calls to develop targeted interventions designed for PBTS and their families that will promote better quality of life and transition preparation [4, 30]. Unfortunately, although PBTS often experience more physical and psychosocial sequelae of their disease relative to other pediatric cancers (Perreault et al., accepted), to date, *no transition readiness intervention has been developed to address the specific disease management, social, and cognitive needs of PBTS.* The current study aims to address this gap by evaluating the feasibility, acceptability, and preliminary efficacy of targeted transition readiness workshops for PBTS which will target disease management, social competence, and cognitive functioning.

### Aims and objectives

Consistent with a model for developing behavior treatments for chronic diseases [31], we will pursue the following aims in the assessment of the pilot targeted transition workshops for PBTS:

#### Aim 1: Testing the feasibility and acceptability of the targeted transition workshops

The primary aim of the current proposal is to assess the feasibility and acceptability of the targeted transition workshops for PBTS. Feasibility will be assessed by reporting on PBTS and caregiver: participation rates (overall recruitment, participation for each workshop theme), satisfaction ratings, and qualitative feedback. Results of this aim will inform the implementation of the workshops in routine clinical practice.

#### Aim 2: Preliminary efficacy testing

We will examine the impact of targeted transition workshop attendance on a preliminary efficacy outcome measure of PBTS transition readiness skills using the TRAQ. We will also explore social competence, cognitive functioning, and parent overprotection as mediators of improvements in transition readiness skills, as well as the impact of intervention participation on adaptive behaviors.

## Methods

### Intervention: targeted transition readiness workshops

Three 1 ½-h workshops will be offered to participants over a 6-month period (at 2-month intervals). Each workshop will cover a specific topic related to PBTS transition readiness skills: (1) disease self-management, (2) social competence, and (3) cognitive functioning (see Table 1). Skills targeted by the intervention were initially chosen based on transition readiness skills identified via the TRAQ, those identified by previous literature as being particularly challenging for PBTS, as well as group consensus during multidisciplinary consultation meetings [28]. Once specific skills were identified, we incorporated, where available, existing intervention strategies (e.g., the 3-sentence health summary approach, SMART goal setting). The intervention also incorporates the Social-ecological Model of Adolescent and Young Adult Readiness to Transition (SMART) by including psychosocial factors (social competence and cognitive functioning) in addition to disease management skills [32]. At each event, concurrent workshops on the same theme will be offered to both PBTS and their caregivers separately to increase PBTS’ skills for their autonomy, and to help caregivers to support PBTS in acquiring these skills. Workshops will be available in person, as well as via an online virtual platform (e.g., Zoom) to accommodate more equitable access for remote participants.

**Table 1:** Targeted transition workshops

	**Workshops for PBTS and caregivers**		
	1st workshop	2nd workshop	3rd workshop
Healthcare professional	Transition care nurse	Social worker/psychologist	Occupational therapist/psychologist
Theme	Disease self-management	Social skills and peer relationships	Cognitive challenges and return to daily activities
Psychoeducation	Differences between pediatric and adult setting	Components of friendship and opportunities for socialization	Overview of school and work resources (education plans, scholarships, funds, work placement programs) and how to access these
Transition skill education and practice	- How to manage medications (filling a prescription, reading medication labels) - How to share a personal health history	- How to ask questions/which questions to ask -How to assert needs [33]	-How to learn and access services for managing daily activities (how to ask for adapted resources at school, work) -How to plan and organize daily activities (SMART goals)

The intervention approach is based on the ABC transition process [34] consisting of awareness (psychoeducation), building capacity (skill building), and collaboration (stakeholder knowledge sharing). The intervention was developed and refined involving consultation with key stakeholders in the pediatric oncology program, including physicians, nurses, social workers, occupational therapists, psychologists, and a parent partner. Ongoing stakeholder consultation is incorporated in all phases of the project (development, implementation, evaluation, and knowledge dissemination). Three discussion meetings between the research team and stakeholders were specifically devoted to refining the content and delivery of each workshop topic (disease self-management, social skills, cognitive functioning). In addition, all workshops will be co-facilitated by a PBTS post-transfer to adult healthcare services (for survivors’ workshops) and by a parent of a post-transfer PBTS (for parents’ workshops). Indeed, the workshop format was selected in order to facilitate the post-transfer survivor and parent reaching a larger group simultaneously (rather than individually), the ability to incorporate the interactive component (dynamic question and answer period rather than pre-recorder responses), and the ability to connect PBTS participants and caregivers in geographically distant locations by using a hybrid workshop approach. See Table 1 for workshop components.

Each workshop will follow the same structure: (1) topic presentation by a healthcare professional and post-transfer PBTS/caregiver, (2) teaching two specific strategies, (3) question and answer period with participants and a post-transfer PBTS or their caregiver (for pre-transfer PBTS and caregiver groups respectively), and (4) workshop evaluation (satisfaction survey and brief focus group).

### Study design (see Fig. 1 for timeline)

**Fig. 1:**
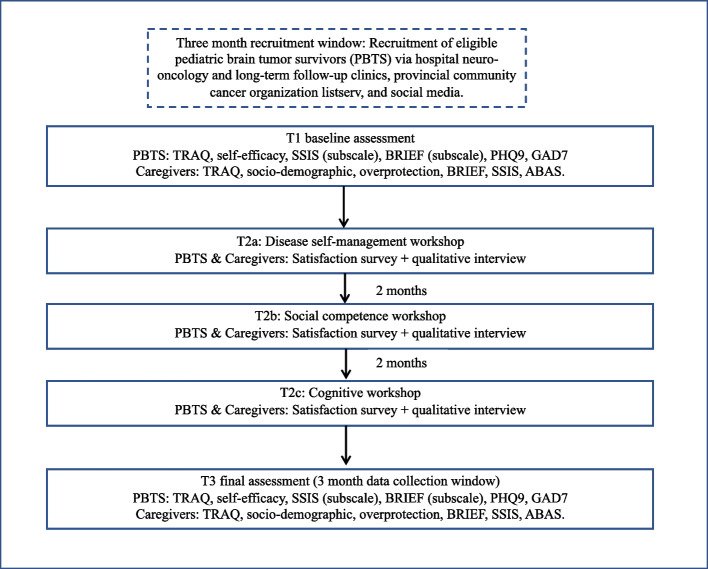
Study procedure/timeline

This is a pre-post feasibility study of the targeted transition workshops for PBTS, based on a mixed convergent method which will include quantitative and qualitative data collection [35]. Baseline data collection (T1) will occur prior to transition workshops. Brief satisfaction and qualitative feedback will be collected at the conclusion of each workshop which will be spaced out at 2-month intervals (T2a, b, c). Final data collection will occur following the completion of transition workshops (T3).

### Participant inclusion/exclusion criteria

Participants will be recruited by dyads, with a caregiver for each eligible youth also being invited to participate in separate parallel caregiver workshops. *Youth inclusion criteria*: PBTS (any cancerous or non-cancerous brain tumor diagnosis), at least 1 year post-active brain tumor treatment (radiation, chemotherapy), 14 years or older, pre-transfer to adult hospital care, French or English speaking. *Inclusion criteria for caregiver*: Primary caregiver of PBTS (as outlined above). Both PBTS and caregiver must agree to participate in the study. *Youth exclusion criteria*: actively receiving treatment for a relapse or palliative care, primarily receiving care in an adult healthcare setting, having one or several significant physical, cognitive, and psychosocial sequelae preventing their participation in the group (as determined by the treating oncologist/nurse), primary caregiver does not participate. *Exclusion criteria for caregiver*: does not self-identify as the primary caregiver; PBTS does not consent to participate.

### Recruitment procedure

This study has been approved by the Institutional Review Board of Sainte-Justine University Health Center. Participants will be recruited over a 3-month window prior to the first workshop (see Fig. 1 for the timeline). Families of eligible PBTS participants will be recruited in three ways:

1. Via* email/online platforms*: A member of the Leucan association, which is a non-profit organization for children with cancer and their families, will make a pre-selection of the families registered for their email listserv according to the diagnosis (brain tumor) and the age of their child (14 years and older). An email will then be sent to eligible families with the flyer containing study information and families interested in participating will be invited to contact the research coordinator. The flyer will also be shared via online platforms (e.g., Brain Tumor Foundation of Canada research page, Sainte-Justine Research Institute social media accounts).
2. *In the Sainte-Justine University Health Center (SJUHN) clinic*: The research project will be presented to patients identified as potential participants by healthcare providers working within the SJUHN neuro-oncology clinic as well as the SJUHN long-term follow-up clinic.
3. *Through an existing database*: Eligible participants may also be identified through a neuro-oncology patient database maintained by the SJUHN brain tumor clinic. The research team will contact eligible participants by phone to inform them of the study.

To be eligible to participate, the PBTS and caregiver must assent/consent (both must participate). Youth will be asked to sign the assent form. However, should an eligible youth present themselves to the workshop without a caregiver, they could consent to participate given their age (over 14 years old), the minimal risks involved in the study, and the potential benefit of attending the workshop. After the assent/consent form is signed, the baseline measures (T1) are completed. Participants will be given the option of completing survey questionnaires in person (e.g., before a clinic appointment) or at home via the online secure platform LimeSurvey (https://www.limesurvey.org). Each participant (PBTS and caregiver in each dyad) receives a $20 gift card at baseline (T1) and completion of parallel measures post-workshops (T3) as a token of appreciation for their participation in the study. We will also provide parking passes or public transit reimbursement for families attending the workshops in person ($10 per family per workshop). Workshop participation will not be compensated in order to better estimate interest in future non-monetized workshop participation.

### Sample size

We estimate recruiting approximately 40 PBTS and caregiver dyads. The sample size is based on recommendations for a pilot feasibility study and will therefore be appropriate for study purposes [36].

### Assessments and outcome measures

The primary outcomes are the feasibility and acceptability of the intervention. A secondary outcome is transition readiness skills. Exploratory analyses will examine the potential changes in parental overprotection, PBTS self-efficacy, assertiveness, planning, organization skills, and adaptive behaviors. Demographic, mental health, and clinical variables are also collected for descriptive purposes.

#### Feasibility and acceptability measures

We will collect frequency data on recruitment rate, number of sessions attended, attendance rate by workshop type, and type of workshop attendance (in person versus virtual). Intervention acceptability will be measured using a short quantitative satisfaction survey and a brief qualitative focus group at the end of each workshop.

##### Satisfaction survey

A self-report Likert scale measure will assess satisfaction with various facets of the workshops (e.g., the length of workshops, skills targeted, format, workshop presenters). There will also be opportunities for participants to provide comments, including what they liked the most and the least regarding workshops.

##### Focus group questions

At the conclusion of each workshop, a brief 30-min exit interview will take place separately with caregivers and youth to obtain open-ended feedback on workshop format and content (e.g., What did you like? What could be done differently?).

#### Preliminary efficacy measures

##### Transition readiness assessment questionnaire

The TRAQ is a questionnaire aimed at measuring adolescent and young adult patients’ transition readiness skills. It comprised 20 items divided into five domains: (1) the management of medications (e.g., filling up prescriptions), (2) appointment keeping (e.g., calling the doctor’s office to schedule an appointment), (3) tracking health issues (e.g., making a list of questions prior to a medical visit), (4) talking with providers (e.g., answering the doctor or the nurse’s questions), and (5) management of daily activities (e.g., helping in planning or preparing meals).

#### Exploratory outcome measures

##### Parent overprotection

The Parent Protection Scale (PPS) will be used to assess parental overprotection [37]. The PPS, a 25-item self-report measure, examines several dimensions of overprotective parenting behaviors. Parents are asked to rate the extent to which each statement is descriptive of their behavior with their child on a 4-point scale ranging from 0 (“never”) to 3 (“always”). Items include: “I let my child make his/her own decisions.” A higher total score indicates a higher level of protective parenting behaviors.

##### Self-efficacy

The General Self-Efficacy Short Scale (GSE) is designed to assess a person’s belief in his/her capacity to manage daily stressors and have control over meaningful events [38]. The measure includes 10 items and uses a Likert scale with frequency response options ranging from “not at all true” to “very true.” Higher scores reflect greater general self-efficacy.

##### SSIS

Social skills will be measured by the Social Skills Improvement System [39]. The Social Skills Rating System (SSRS) provides an age and gender-normed total standard score representing four subscale scores: cooperation, assertion, self-control, and responsibility. To reduce the burden on PBTS, caregivers will complete the full measure, while PBTS will complete only the assertiveness scale items. The SSIS has adequate reliability and validity, and compared to other measures used to assess social competence, SSRS has the most comprehensive data within pediatric brain tumor survivors [40]. Lower scores on the total scale reflect greater problems in social skills.

##### BRIEF

The Behavior Rating Inventory of Executive Function (BRIEF) was designed to capture an individual’s executive functioning capabilities within a real-world context through the use of an informant report [41]. The executive functioning framework used by this measure includes eight clinical scales: Inhibit—resist impulses; Shift—adjust allocation of attention and transition between tasks; Emotional Control—regulate and modulate emotion; Initiate—start tasks; Working Memory—hold information in one’s immediate awareness long enough to perform a given task; Plan/Organization—use future orientation to complete steps in a sequence to meet a goal; Organization of Materials—effectively manage belongings; and Monitor—self-check one’s progression with a task and adjust accordingly. Higher scores reflect greater problems in executive function abilities. To reduce the burden on PBTS, caregivers will complete the full measure, while PBTS will complete only the Plan/Organization scale items.

##### ABAS

The Adaptive Behavior Assessment System-Second Edition (ABAS-II) Parent Form is a widely used, caregiver-completed questionnaire that assesses adaptive behavior in individuals aged 5–21 years [42]. Caregivers rate their child’s ability to perform daily tasks correctly when needed. It consists of nine subscales that form a Conceptual composite, a Social composite, and a Practical composite. The Conceptual composite comprises Communication, Self-direction, and Functional Academics subscales and is used to assess skills such as conversational turns, the ability to work independently, and keeping lists or reminders. The Social composite comprises the Leisure and Social subscales and is used to assess skills such as waiting turns and listening to others. The Practical composite comprises the Self-Care, Home Living, Health/Safety, and Community Use subscales and is used to assess skills such as rules for community safety, maintaining household duties, and finding public restrooms. The ABAS-II also yields a Global composite of overall adaptive functioning, the Global Adaptive Composite. The ABAS-II has demonstrated high internal consistency (*r* values range from 0.85 to 0.99) and high test–retest reliability (*r* values range from 0.80 to 0.90) [42].

#### Sample descriptive measures

##### Sociodemographic survey

We will use a short questionnaire (13 items) with PBTS and their parents in order to collect sociodemographic information (date of birth, sex, place of residence, family situation, education level and work status) as well as clinical information (type of tumor, history of the disease, and its treatments) which will allow us to establish a general profile of the participants in this study. Moreover, the information gathered with this measure will be possibly used as a covariate if the sample size will be sufficient, to evaluate the impact of socioeconomic status on transition readiness.

##### PHQ-9

The Patient Health Questionnaire (PHQ-9) is a widely used self-report screening tool for depression based on the Diagnostic and Statistical Manual of Mental Disorders-IV (DSM-IV) criteria [43]. The tool consists of 9 items that assess whether the symptoms have bothered the individual during the previous 2 weeks. The summed score ranges from 0 to 27 and can be categorized into 4 categories: minimal (0 ~ 4), mild (5 ~ 9), moderate (10 ~ 14), and severe (≥ 15). The psychometrics are considered strong, with good reliability and validity [43].

##### GAD-7

The General Anxiety Disorder (GAD-7) has been shown to be a valid and effective measure of anxiety in the general population [44]. The item scales of this measure, based on DSM-IV criteria, focus on the presence of seven core anxiety symptoms in the last 2 weeks. The total scores range between 0 and 21 and can also be categorized into four categories: minimal (0 ~ 4), mild (5 ~ 9), moderate (10 ~ 14), and severe (≥ 15). The psychometrics are also considered strong, with good reliability and validity [45].

### Data analysis

#### Aim 1: Feasibility and acceptability testing

Uptake will be measured by descriptively reporting: recruitment rate for those who accept to attend the three workshops (percentage), attendance rate at each workshop, and participation rate of in person versus online. The workshops will be considered feasible if ≥ 60% of recruited participants participate in at least 2 workshops. *Satisfaction* will be measured using a Likert questionnaire assessing satisfaction with content and format. Consistent with an evaluation of other healthcare interventions, the workshops will be deemed acceptable if ≥ 75% of participants report workshops to be “acceptable” or “very acceptable” on the Satisfaction Survey [46].

*Qualitative feedback* will consist of analyzing workshop exit interviews. All focus groups will be audio-recorded and transcribed. Consistent with our previous experience in intervention refinement [47], we will use an inductive thematic analysis approach and aided by the MAXQDAA software [48]. Thematic analysis includes the following phases: data familiarization (phase 1), initial coding (phase 2), searching for themes (phase 3), refinement of themes (phase 4), defining and naming themes (phase 5), and report (phase 6).

#### Aim 2: Preliminary efficacy testing

We will conduct a paired samples *T* test to compare transition readiness skills between PBTS pre- and post-workshop participation. We will also conduct an ANCOVA to examine the impact of workshop attendance (0–3 sessions) on TRAQ skills post-workshop, controlling for pre-workshop TRAQ skills.

### Exploratory aims

We will also explore assertiveness, plan/organization, and parent overprotection as mediators of improvements in transition readiness skills, as well as the impact of intervention participation on adaptive behaviors (via examination of effect size changes [Cohen’s *d*] pre/post-workshop participation). This will inform measure selection for a future larger multi-site study of the targeted transition readiness workshops.

### Dissemination

The following knowledge translation activities will be conducted with the intention of advancing the field of transition care for PBTS: delivery of scientific rounds at the participating center to disseminate relevant results for stakeholders in pediatric oncology as well as other pediatric chronic conditions, participation in community presentations for parent organizations, and scientific presentations nationally and internationally. We will also be recording the didactic de-identified portion of the workshops in order to be able to share this with PBTS, their caregivers, and providers more broadly.

## Discussion

This is the first pilot feasibility study of transition intervention workshops targeted for pediatric brain tumor survivors (PBTS) and their parents. It aims to respond to the call in recent literature for the development and validation of interventions supporting the specific transition readiness needs of PBTS. We will develop, deliver, and assess the feasibility and preliminary efficacy of transition skill workshops aimed at PBTS as well as helping parents support the transition and the autonomy of their children towards adult environments. To achieve and evaluate those objectives, we organized a three-step study as follows. (1) The pre-post evaluation with the TRAQ questionnaire will assess the relevance of interventions during the transition period specific to PBTS. (2) The workshops will address sensitive topics that are part of the challenges that PBTSs have to face. (3) The focus groups following the workshops will allow us to collect information from PBTS and their parents in order to better adjust future workshops according to their needs and priorities.

We anticipate that participant recruitment will be acceptable given the previously expressed desire for this type of content, the format (in person and online), and current limited availability of transition interventions specific to the PBTS population. The SJUHN hosts the largest Division of Pediatric Oncology in Quebec and is the primary center for PBTS treatment in the province, thereby facilitating participant recruitment. Further, as a result of improved survival outcomes, there is a growing population of survivors. We hypothesize this sizeable population, our partnership with Leucan (provincial community cancer organization) and the hybrid (in person and virtual) format of workshops will all allow for feasible recruitment of participants. We aim to recruit approximately 40 dyads (one primary caregiver and youth) over a 3-month period. It is expected, however, that participant attrition will occur over the course of the intervention, given the number of workshops. Given the participation of only one primary caregiver, for each workshop, we will transfer the documents created for this purpose, such as the slides presented to the participants as well as a list of resources related to the topic of the workshop, so that it can be subsequently shared with other members of the family. Information on which workshop topics generate the greatest interest and engagement will also be of use to further intervention/resource development.

We anticipate session content and format will be acceptable, particularly the combination of healthcare provider and post-transfer survivor implication. It is expected that the acceptability of the targeted workshops will be moderate to good, considering that this is a new intervention and that the needs and expectations of the participants in terms of preparation for the transition cannot all be met in three workshop sessions. Moderate to good acceptability will allow the workshops to be revised and improved. We hypothesize that those participating will improve their transition readiness skills. Should there be no significant impact, we will use the qualitative data to reformulate workshop content and format for greater impact. Finally, regarding knowledge translation, all workshops will be video-recorded, with the possibility of subsequently sharing didactic content more broadly following study completion.

Ultimately, we hope this intervention will have a positive impact on long-term transition outcomes, such as adherence to adult healthcare appointments. Here, we focus on the preliminary phases of the ORBIT model for intervention development by first testing the feasibility and acceptability of the novel intervention in a small sample [31]. It is likely that the feedback obtained will lead to some changes to the intervention. Our aim is to subsequently test a refined version more conclusively in a larger study, with a longitudinal design to examine the impact on relevant longer-term goals such as age at transfer to adult care and adherence to adult healthcare appointments. A larger sample would also allow for a greater understanding of who may most/least benefit from the intervention and why. Given the presence of disease self-management, social, and cognitive challenges across many pediatric chronic illness populations (e.g., sickle cell, congenital heart disease), we also aim to evaluate the impact of the intervention trans-diagnostically in the future [49]. In addition, we recognize that transition skill development needs to occur in multiple settings and includes continued work on these skills once in adult healthcare settings. Furthermore, here, we focus on supporting patients and caregivers. However, additional important stakeholders in this process are the pediatric and adult healthcare providers who interact with these adolescents and young adults. An abbreviated version of this workshop (written summary, in-person workshop, webinar) could be developed to address provider transition information needs. Adult providers may also consider using the TRAQ as a screening tool to assess transition readiness skills in their young adult patients. Overall, this foundational study will provide much-needed insights for the development of a strong program of transition intervention research which would positively impact many families.

## Data Availability

The data that supports the findings of this study are available from the principal authors, MB and LD, upon reasonable request.

## Abbreviations

PBTS: Pediatric brain tumor survivors
TRAQ: Transition Readiness Assessment Questionnaire
SMART: Social-ecological Model of Adolescent and Young Adult Readiness to Transition
SJUHN: Sainte-Justine University Health Center
PPS: Parent Protection Scale
GSE: General Self-Efficacy Short Scale
SSIS: Social Skills Improvement System
SSRS: Social Skills Rating System
BRIEF: Behavior Rating Inventory of Executive Function
ABAS-II: Adaptive Behavior Assessment System-Second Edition
PHQ-9: Patient Health Questionnaire
DSM-IV: Diagnostic and Statistical Manual of Mental Disorders-IV
GAD-7: General anxiety disorder
